# Survival Benefits and Less Intensive Treatment for Women with Early-Stage Breast Cancer Diagnosed While Participating in Population-Based Screening

**DOI:** 10.1245/s10434-025-17845-1

**Published:** 2025-07-25

**Authors:** Melissa Edwards, Kenneth Elder, Allison Rose, Elizabeth Tan, Allan Park, Carolyn Nickson, G. Bruce Mann

**Affiliations:** 1 Peter MacCallum Cancer Centre, Melbourne, Australia; 2 The Royal Melbourne Hospital, Melbourne, Australia; 3 The University of Auckland, Auckland, New Zealand; 4 Western General Hospital, Edinburgh, UK; 5 University of Melbourne, Melbourne, Australia; 6 The Daffodil Centre, a joint venture between the University of Sydney & Cancer Council NSW, Sydney, Australia

## Abstract

**Background:**

The authors previously reported that within a patient cohort from Victoria, Australia, women who had early-stage breast cancer (ESBC) diagnosed while participating in screening [active screeners (AS), comprising both screen-detected and interval cancers] receive less intensive treatment than those not recently screened (NRS). This study reports mortality and subsequent cancer events for that cohort.

**Methods:**

Follow-up data were collected for 766 (97.1%) of the 789 women in the original cohort (612 [79.9%] AS and 154 [20.1%] NRS), with a median follow-up time of 11.6 years (interquartile range [IQR], 9.8–13.8 years). Mortality and subsequent cancer diagnosis data were derived from linkage with the Victorian Cancer Registry. Breast cancer-specific survival (BCSS) and overall survival (OS) were compared between groups, with sensitivity analyses for potential overdiagnosis and lead time bias.

**Results:**

The 10-years BCSS was 95.4% (95% confidence interval [CI], 93.2–96.8%) for AS versus 86.4% (95% CI 79.7–91.0%) for NRS (hazard ratio [HR], 0.28; 95% CI 0.17–0.48; *p* < 0.001). A survival benefit persisted after adjustment for estimated overdiagnosis (HR 0.38; 95% CI 0.21–0.66; *p* = 0.001) and lead time bias (HR 0.33; 95% CI 0.19–0.58; *p* < 0.001). The 10-year OS also was superior for AS, at 90.6% (95% CI 87.9–92.7%) compared with 82.5% (95% CI 75.4–87.8%) for NRS (HR 0.54; 95% CI 0.36–0.79; *p* = 0.002).

**Discussion:**

Patients who have ESBC diagnosed while participating in screening experience improved BCSS and OS while receiving less intensive treatment. These findings are robust to adjustment for potential overdiagnosis and lead time bias. As treatment for ESBC becomes more tailored, with emerging opportunities for reduced treatment intensity, the benefits of screening are likely to improve further.

**Supplementary Information:**

The online version contains supplementary material available at 10.1245/s10434-025-17845-1.

In high-income settings, reduced breast cancer mortality in recent decades has been attributed to the introduction of population-based mammographic screening programs combined with advancements in adjuvant systemic therapies.^1^ The principle of screening is to advance the diagnosis of breast cancer so that prognosis may be improved by earlier intervention.

The seminal trials of mammographic screening in the 1970s and 1980s showed an overall 20% reduction in breast cancer death among invited women,^2–4^ with the greatest value demonstrated in the 50–69-years age group.^5–7^ More contemporary trial updates and observational analyses have continued to show a significant breast cancer-specific survival (BCSS) benefit from participation in screening.^5,7–9^

Overdiagnosis is the detection of histologically confirmed breast cancer through screening that would never have been diagnosed clinically during the lifetime of the woman. Subsequent overtreatment of clinically insignificant lesions is therefore a potential harm of screening,^3,8,10^ with some commentaries making the implicit assumption that all patients with early-stage breast cancer (ESBC) receive similar comprehensive treatment.^10^ This has led to the suggestion that any benefits of screening from earlier treatment of significant disease may be offset by harms due to unnecessary treatment of lesions never destined to become clinically apparent.^8,11,12^

We previously analyzed treatment patterns in a cohort of women with a diagnosis of ESBC according to their participation in a population-screening program.^13^ The patients were classified as active screeners (AS), which included both screen-detected and interval cancers (defined as cancer diagnosed within 27 months after a negative screen) or as not recently screened (NRS), encompassing the remainder of patients who had either never attended state-based screening, or whose last screening study was more than 27 months before diagnosis.

We compared the extent of treatment according to screening participation, finding that AS patients underwent fewer mastectomies and axillary dissections and received less adjuvant chemotherapy and post-mastectomy radiotherapy. These findings persisted after adjustment for potential overdiagnosis. This analysis was unable to determine the associations between earlier diagnosis and mortality outcomes, or to address the possibility that increasingly effective systemic therapies may offset mortality risks related to later diagnosis.

The current study assessed the survival and subsequent cancer event outcomes for this cohort of patients according to screening status at their original diagnosis.

## Methods

The original study cohort comprised 789 women ages 50–69 years (the historical target age range for breast cancer screening in Australia) with incident ESBC (invasive or ductal carcinoma in situ [DCIS]) newly diagnosed between 2007 and 2013 and treated within a single breast service (the Royal Melbourne and Royal Women’s Hospitals, Victoria, Australia). Screening status was ascertained through linkage with BreastScreen Victoria records via the Victorian Cancer Registry (VCR). Methodology pertaining to the ascertainment of patient and disease characteristics as well as treatment received/recommended has been described previously,^13^ with records maintained for the current analysis. 

Mortality data were obtained through updated linkage of the cohort with the VCR, which routinely collects death records from the Victorian Registry of Births, Deaths and Marriages, as well as the National Deaths Index, coding underlying cause of death in accordance with the World Health Organisation International Classification of Diseases guidelines for mortality coding. Data extraction was performed on 2 November 2023, with event data censored as of 31 December 2022 (the most recent date at which VCR mortality data were considered complete at the time of extract).

Follow-up data were available for 766 (97.1%) of the 789 patients in the original cohort, with 21 patients (2.65%) unable to be linked with registry data and 2 patients discovered to have metastatic disease within 6 months after their breast cancer diagnosis, and therefore retrospectively considered not to have had early-stage disease at diagnosis.

Subsequent cancer event data also were derived from the VCR and manually cross-referenced with hospital medical and multidisciplinary meeting records. Ipsilateral and contralateral diagnoses longer than 6 months after the initial breast cancer diagnosis were considered to be new cancer events. We were unable to formally report on metastatic recurrence events because their data are poorly captured by the VCR, which requires histologic proof of cancer relapse, so sole radiologic diagnoses are likely to be missed.

Ethical approval for data linkages and analyses was obtained from the Cancer Council Victoria Human Research Ethics Committee (approval no. 1515), with additional research approvals from the BreastScreen Victoria Research Committee and the Melbourne Health Research and Ethics Committee.

### Statistical Analysis

The primary outcome for the analysis was BCSS, with overall survival (OS), first ipsilateral breast events, and first contralateral breast events as secondary outcomes of interest.

Descriptive statistics for study variables were computed using complete cases, with comparison of categorical variables by screening status (NRS vs AS) using chi-square tests. Of the 789 cases, 190 (24.8%) had at least one missing variable, with the majority of case-wise missingness driven by human epidermal growth factor receptor 2 (HER2) status, predominately due to incomplete reporting of HER2 status for cases in which the dominant lesion was pure DCIS (missing in 18.2% overall, although in only 0.99% of the cases with invasion). Missing values analysis was compatible with a missing-at-random mechanism. A multiple imputation model was thus applied using chained equations producing 40 imputed datasets, with results combined using Rubin’s rules.^14^

Associations between screening status and survival were assessed using Kaplan-Meier survival methods and Cox proportional hazards models, producing hazard ratios (HRs) and 95% confidence intervals (CIs), together with predicted survival curves. Individuals who remained event-free at the conclusion of the study follow-up period were censored as of 31 December 2022. Multiply imputed and complete case estimates for Cox models were similar, with imputed estimates presented. Absolute differences in survival by screening status at 5, 10, and 15 years were estimated using complete cases, with before and after adjustment for age at diagnosis and tumor characteristics assumed to be unchanged by earlier diagnosis (grade, estrogen receptor [ER], progesterone receptor [PR], and HER2 status).

As applied in the previous study of this cohort, a sensitivity analysis was performed to correct for potential overdiagnosis by excluding all screen-detected DCIS and any screen-detected node-negative, grade 1, < 10-mm, ER- and/or PR-positive, HER2-negative invasive cancers. Additional sensitivity analysis also was performed for BCSS to assess potential lead time bias, applying a 40-months advancement in diagnosis time for screen-detected cancers,^15^ thereby reducing the estimated survival associated with those diagnoses.

Statistical analysis was performed using Stata/IC for Macintosh, version 14.2 (StataCorp LLC, College Station, TX). All tests of significance were two-tailed, and *p* values lower than 0.05 were considered statistically significant.

## Results

Of the 766 patients included in this follow-up study, 621 (79.9%) were designated as AS (with 560 [73.1%] screen-detected and 52 [6.8%] interval cancers) and 154 (20.1%) as NRS. Demographic, disease, and treatment characteristics of the cohort together with event rates are shown in Table 1. Indigenous (Aboriginal or Torres Strait Islander) ethnicity accounted for 0.65% of the study population. The majority of the tumors (79.4%) were invasive. Of the 158 patients with pure DCIS, 82 (51.9%) had high-nuclear-grade tumors. The women presenting NRS were more likely to have invasive, higher-grade, larger-size, node-positive HER2-positive tumors. Among screen-detected cancers, 10.2% were HER2-positive, whereas 5.7% were of the triple-negative phenotype, compared with 16.7% HER2-positive/14.6% triple-negative tumor for interval cancers and 17.5% HER2 positive/8.8% triple-negative tumor for NRS. Of the 560 screen-detected cases, 180 (32.1%) met our definition of potential overdiagnosis (137 [24.5%] *in situ*, 43 [7.7%] invasive). The characteristics of the potentially overdiagnosed cases are displayed in Table S1.

**Table 1:** Characteristics of the study cohort

	Overall		Screen-detected		Interval		All AS		NRS		*p* Value^a^
	*n*	%	*n*	%	*n*	%	*n*	%	*n*	%	
*n*	766	–	560	73.1	52	6.79	612	79.9	154	20.1	
*Age (years)*											
50–59	383	50.0	266	47.5	27	51.9	293	47.9	90	58.4	0.02
60–69	383	50.0	294	52.5	25	48.1	319	52.1	64	41.6	
*Menopausal status*											
Pre-/peri-menopausal	96	12.5	68	12.1	7	13.5	75	12.3	21	13.6	0.14
Post-menopausal	543	70.9	407	72.7	36	69.2	443	72.4	100	64.9	
Unknown	127	16.6	85	15.2	9	17.3	94	15.4	33	21.4	
*Invasive/in situ*											
Invasive	608	79.4	423	24.5	48	7.69	471	77	137	89	0.001
In situ	158	20.6	137	75.5	4	92.3	141	23	17	11	
*Size (invasive) (mm)*											
Median (IQR)	15 (9.5–23)		13 (8–19)		24 (17.5–30)		14 (8–20)		21 (14–30)		<0 .001
<10	152	25.0	130	30.7	6	12.5	136	28.9	16	11.7	
10–19	239	39.3	188	44.4	9	18.8	197	41.8	42	30.7	
20–29	122	20.1	65	15.4	16	33.3	81	17.2	41	29.9	
≥30	95	15.6	40	9.46	17	35.4	57	12.1	38	27.7	
*Size (in situ) (mm)*											
Median (IQR)	14 (6–27)		15 (7–27)		14.5 (2.5–47.5)		10 (2–24)		15 (6.5–27)		0.85
<10	57	36.1	47	34.3	2	50.0	49	34.8	8	47.1	
10–19	36	22.8	32	23.4	0	0	32	22.7	4	23.5	
20–29	29	18.4	26	19.0	1	25.0	27	19.1	2	11.8	
≥30	35	22.2	31	22.6	1	25.0	32	22.7	3	17.6	
Unknown	1	0.63	1	0.73	0	0	1	0.71	0	0	
*Grade (invasive)*											
1	128	21.1	108	25.5	9	18.8	117	24.8	11	8.03	< 0.001
2	234	38.5	168	39.7	14	29.2	182	38.6	52	38.0	
3	196	32.2	112	26.5	24	50.0	136	28.9	60	43.8	
Unknown	50	8.22	35	8.27	1	2.08	36	7.64	14	10.2	
*Grade (in situ)*											
Low	25	15.8	21	15.3	0	0	21	14.9	4	23.5	0.28
Intermediate	48	30.4	45	32.8	1	25.0	46	32.6	2	11.8	
High	82	51.9	69	50.4	2	50.0	71	50.4	11	64.7	
Unknown	3	1.90	2	1.46	1	25.0	3	2.13	0	0	
*Positive nodes (invasive)*											
0	418	68.8	325	76.8	23	47.9	348	73.9	70	51.1	< 0.001
1–3	123	20.2	78	18.4	11	22.9	89	18.9	34	24.8	
4–10	43	7.07	14	3.31	10	20.8	24	5.1	19	13.9	
>10	19	3.13	6	1.42	3	6.25	9	1.91	10	7.3	
Unknown	5	0.82	0	0	1	2.08	1	0.21	4	2.92	
*ER status*											
Positive	649	84.7	492	87.9	35	67.3	527	86.1	122	79.2	0.09
Negative	103	13.4	58	10.4	16	30.8	74	12.1	29	18.8	
Unknown	14	1.83	10	1.79	1	1.92	11	1.80	3	1.95	
*PR status*											
Positive	568	74.2	431	77.0	32	61.5	463	75.7	105	68.2	0.15
Negative	183	23.9	118	21.1	19	36.5	137	22.4	46	29.9	
Unknown	15	1.96	11	1.96	1	1.92	12	1.96	3	1.95	
*HER2 status (invasive)*											
Positive	75	12.3	43	10.2	8	16.7	51	10.8	24	17.5	0.03
Negative	527	86.7	377	89.1	40	83.3	417	88.5	110	80.3	
Unknown	6	0.99	3	0.71	0	0	3	0.64	3	2.19	
*Triple-negative (invasive)*											
No	557	91.6	395	93.4	41	85.4	436	92.6	121	88.3	0.11
Yes	43	7.07	24	5.67	7	14.6	31	6.58	12	8.76	
Unknown	8	1.32	4	0.95	0	0	4	0.85	4	2.92	
*Breast surgery*											
BCS	611	79.8	468	83.6	38	73.1	506	82.7	105	68.2	< 0.001
Mastectomy	155	20.2	92	16.4	14	26.9	106	17.3	49	31.8	
*Axillary surgery*											
SLNB	516	67.4	398	71.1	33	63.5	431	70.4	85	55.2	< 0.001
ALND (± SLNB)	149	19.5	74	13.2	16	30.8	90	14.7	59	38.3	
None	101	13.2	88	15.7	3	5.77	91	14.9	10	6.49	
*Radiation therapy*^*b*^											
No	200	26.1	159	28.4	6	11.5	165	27.0	35	22.7	0.29
Yes	566	73.9	401	71.6	46	88.5	447	73.0	119	77.3	
*Chemotherapy*^*b*^											
No	504	65.8	420	75.0	18	34.6	438	71.6	66	42.9	< 0.001
Yes	262	34.2	140	25.0	34	65.4	174	28.4	88	57.1	
*Endocrine therapy*^*b*^											
No	161	21.0	109	19.5	15	28.8	124	20.3	37	24.0	0.31
Yes	605	79.0	451	80.5	37	71.2	488	79.7	117	76.0	
*Subsequent cancer events*											
Ipsilateral breast events	39	5.09	28	5.00	3	5.76	31	5.07	8	5.19	0.95
Contralateral breast events	43	5.61	29	5.18	4	7.69	33	5.39	10	6.49	0.60
*Deaths*											
Breast cancer	55	7.18	25	4.46	5	9.62	30	4.90	25	16.2	< 0.001
Other cancer	22	2.87	17	3.04	0	0	17	2.78	5	3.25	0.38
Non-cancer	39	5.09	31	5.54	3	5.78	34	5.56	5	3.25	0.003
Unknown	2	0.26	1	0.18	0	0	1	0.16	11	7.14	0.55

*AS* active screener, *NRS* not recently screened, *IQR* interquartile range, *ER* estrogen receptor, *PR* progesterone receptor, *HER2* human epidermal growth factor receptor 2, *BCS* breast-conserving surgery, *SLNB* sentinel lymph node biopsy, *ALND* axillary lymph node dissection

^a^AS vs NRS group comparison by chi-square tests

^b^Treatment recommendation from multidisciplinary meeting

The median follow-up time was 11.6 years (interquartile range [IQR], 9.8–13.8 years). Overall, 118 (15.4%) of the 766 patients in the cohort died, with 55 (46.6%) deaths due to breast cancer, 22 (18.6%) from other cancers, and 39 (33.1%) non-cancer related. The cause of death for two patients was unknown. Five breast cancer-related deaths occurred among the patients who met the study criteria for potential overdiagnosis.

**Fig. 1:**
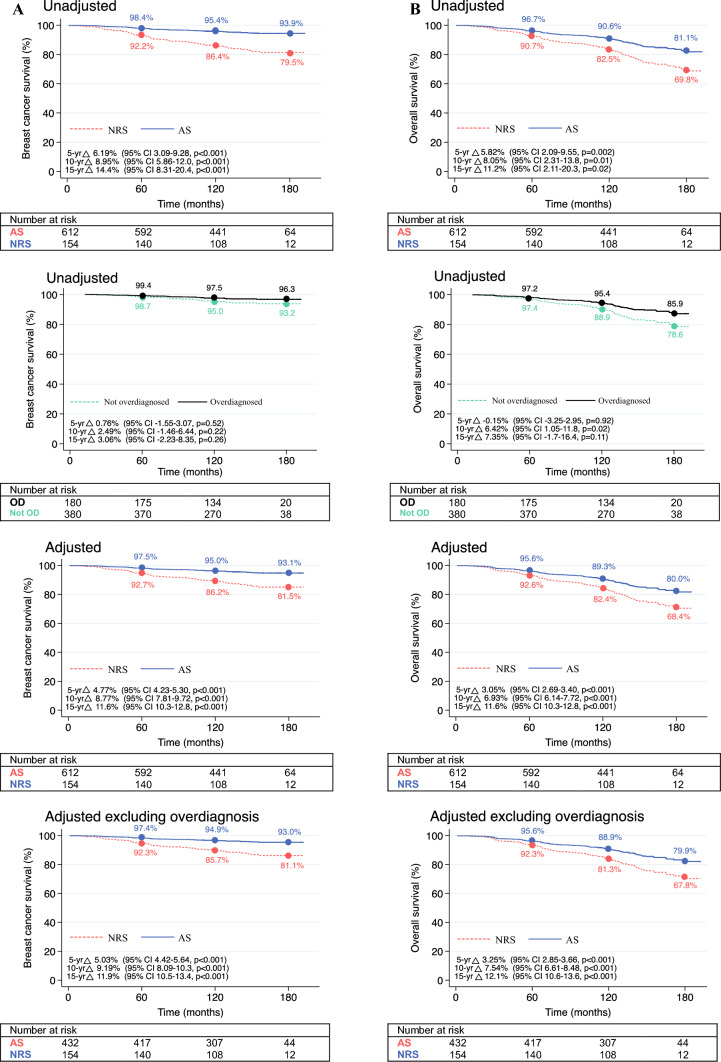
Predicted breast cancer-specific and overall survival curves by screening status. **A** Breast cancer-specific survival. **B** Overall survival. Survival curves predicted from Cox proportional hazards models using complete cases. Adjusted models accounting for age, grade, ER status, PR status, and HER2 status. Overdiagnosis defined as screen-detected DCIS or screen-detected grade 1, < 10-mm, ER-positive/HER2-negative invasive cancers. Comparison with not overdiagnosed screen-detected cases. ER, estrogen receptor; PR, progesterone receptor; HER2, human epidermal growth factor receptor 2; DCIS, ductal carcinoma *in situ;* AS, active screener; CI, confidence interval; NRS, not recently screened; yr, year; Δ, difference

Unadjusted 10-year BCSS was superior for the AS patients at 95.4% (95% CI 93.2–96.8%) versus 86.4% (95% CI 79.7 –91.0%) for the NRS patients (HR, 0.28; 95% CI 0.17–0.48; *p* < 0.001; Fig. 1; Table 2). This BCSS benefit persisted after adjustment for age/disease characteristics and correction for estimated overdiagnosis (10-year difference: 9.19% [95% CI 8.1–10.3%; *p* < 0.001]; HR, 0.38 [95% CI 0.21–0.66; *p* = 0.001]), and after accounting for lead time bias (10-year difference: 8.9% [95% CI 7.9–9.9%; *p* < 0.001]; HR, 0.33 [95% CI 0.19–0.58; *p* < 0.001]).

**Table 2:** Cox proportional hazards regression for breast cancer-specific and overall mortality by screening status

	Breast cancer mortality		Overall mortality	
	HR (95% CI)	*p* Value	HR (95% CI)	*p* Value
Screening status	*Unadjusted*			
NRS	Ref		Ref	
All AS	0.28 (0.17–0.48)	0.01	0.54 (0.36–0.79)	0.002
Interval	0.58 (0.22–1.51)	0.26	0.63 (0.29–1.35)	0.23
Screen-detected	0.26 (0.15–0.45)	<0.001	0.53 (0.35–0.76)	0.002
	Adjusted^a^			
NRS	Ref		Ref	
All AS	0.30 (0.18–0.53)	<0.001	0.53 (0.35–0.79)	0.002
Interval	0.58 (0.33–1.54)	0.28	0.68 (0.31–1.46)	0.32
Screen-detected	0.28 (0.16–0.49)	<0.001	0.52 (0.34–0.78)	0.002
	Adjusted excluding overdiagnosis^a,b^			
NRS	Ref		Ref	
All AS	0.38 (0.21–0.66)	0.001	0.61 (0.40–0.93)	0.02
Interval	0.60 (0.23–1.57)	0.30	0.68 (0.32–1.48)	0.33
Screen-detected	0.36 (0.20–0.65)	0.001	0.60 (0.39–0.92)	0.02

*HR* hazard ratio, *CI* confidence interval, *NRS* not recently screened, *Ref* reference category, *AS* active screener, *ER* estrogen receptor, *PR* progesterone receptor, *HER2* human epidermal growth factor receptor 2, *DCIS* ductal carcinoma in situ

^a^Model adjusted for age, grade, ER status, PR status, and HER2 status

^b^Overdiagnosis defined as screen-detected DCIS or screen-detected grade 1, < 10 mm, ER- and/or PR-positive, HER2-negative invasive cancers. *n* = 586

Multiply imputed estimates

Overall survival also was superior for the AS patients, with an unadjusted 10-year OS rate of 90.6% (95% CI 87.9–92.7%) compared with 82.5% (95% CI 75.4–87.8%) for the NRS patients (HR, 0.54; 95% CI 0.36–0.79; *p* = 0.002; Fig. 1, Table 2). This survival benefit persisted after model adjustment for age and disease characteristics and estimated overdiagnosis (10-year difference: 7.5%; [95% CI 6.6–8.5%; *p* < 0.001]; HR, 0.61 [95% CI 0.40–0.93; *p* = 0.02]).

For the AS patient group, the BCSS and OS benefits were largely due to improved survival among the patients with screen-detected cancers, with no discernible difference in survival between the AS patients with a diagnosis of interval cancer and the NRS patients (Table 2). Among the screen-detected patients, BCSS did not differ between the potentially overdiagnosed cases and those not overdiagnosed (HR, 0.51; 95% CI 0.19–1.37; *p* = 0.19). Overall survival was marginally superior for the patients with potential overdiagnosis, but with a 10-year OS rate of 95.4% (95% CI 90.9–97.7%) compared with 88.9% (95% CI 85.2–91.8) for the patients with non-overdiagnosed screen-detected disease (HR, 0.57; 95% CI 0.33–0.995; *p* = 0.048) (Fig. 1).

Of the 766 patients, 39 (5.1%) experienced a subsequent ipsilateral diagnosis, and 32 (82.1%) of these were invasive events. A contralateral breast event developed in 43 (5.6%) of the 766 patients, 31 (72.1%) of which were invasive. No significant differences between the hazard of first ipsilateral (HR, 0.91; 95% CI 0.42–1.99; *p* = 0.82) and the hazard of first contralateral (HR, 0.76; 95% CI 0.38–1.55; *p* = 0.46) breast events were noted by screening status (Table 3).

**Table 3:** Cox proportional hazards regression for ipsilateral and contralateral breast events by screening status

	Ipsilateral breast events		Contralateral breast events	
	HR (95% CI)	*p* Value	HR (95% CI)	*p* Value
Screening status	*Unadjusted*			
NRS	Ref		Ref	
All AS	0.91 (0.42–1.99)	0.82	0.76 (0.38–1.55)	0.46
Interval	1.06 (0.28–4.01)	0.09	1.15 (0.36–3.67)	0.81
Screen-detected	0.90 (0.41–1.98)	0.79	0.73 (0.36–1.50)	0.39
	*Adjusted*^*a*^			
NRS	Ref		Ref	
All AS	0.92 (0.41–2.04)	0.83	0.78 (0.38–1.62)	0.51
Interval	1.10 (0.29–4.18)	0.89	1.08 (0.34–3.50)	0.89
Screen-detected	0.9 (0.40–2.02)	0.80	0.75 (0.36–1.57)	0.44

*HR* hazard ratio, *CI* confidence interval, *NRS* not recently screened, *Ref* reference category, *AS* active screener, *ER* estrogen receptor, *PR* progesterone receptor, *HER2* human epidermal growth factor receptor 2

^a^Model adjusted for age, grade, ER status, PR status and HER2 status

Multiply imputed estimates

## Discussion

This study demonstrated that in addition to receiving less intensive treatment, the cohort of patients participating in population-based screening, when the diagnosis was ESBC, had better long-term survival than the patients not actively participating. We found that the AS patients had a 72% lower hazard of breast cancer death during a median 11.6-year follow-up period, which translated to an absolute 9% (95% CI 5.9–12.0%) higher rate of breast cancer survival 10 years after diagnosis. Adjustment for patient age, tumor grade, and receptor status made a minimal difference to this estimate, with the findings robust to adjustments for overdiagnosis and lead time bias, confirming that in this patient cohort, earlier detection through screening not only reduced the intensity of treatment required, but substantially improved long-term survival.

Since the time of the original screening trials, breast cancer survival has improved among both screen-detected and symptomatic tumors. Advances in systemic therapy has led some commentators to question the benefit of screening and its ongoing relevance to population health in the modern era.^11,16^ Our findings show that the association between earlier diagnosis and mortality outcomes persists in the context of contemporary systemic therapy.

In addition to improved survival, diagnosis of breast cancer at an earlier stage through screening provides an opportunity for less intensive treatment, with socioeconomic benefits and markedly reduced morbidity for the increasing proportion of patients who go on to live for many years after treatment.^17^ Studies conducted within modern-service Australasian screening programs have reported lower numbers of mastectomies and axillary dissections among women participating in screening, as well as less chemotherapy and immunotherapy.^13,18,19^ In keeping with the increased rates of breast conservation, women with breast cancer diagnosed via screening tend to receive more radiotherapy overall,^18^ but the rates of post-mastectomy radiotherapy are significantly lower.^13,18^

Recent advances in the locoregional treatment of ESBC have provided opportunities for de-escalation, with some aspects of previously standard therapy omitted for patients with very-early-stage disease. For example, evolving evidence from the SOUND^20^ and INSEMA^21^ trials indicate that surgical staging of the axilla with sentinel lymph node biopsy may be safely spared for patients with clinically node-negative T1-2 tumors, for which pathologic nodal status is not required to inform adjuvant therapies.

A growing body of evidence is supportive of omitting radiation completely for certain older adults with favorable disease,^22–24^ with multiple ongoing trials attempting to use molecular phenotypes^25–28^ or preoperative MRI staging^29^ to identify a group of low-risk patients for whom radiotherapy may safely be omitted. The viability of single-method radiotherapy or endocrine therapy after breast-conserving surgery for older adults with stage 1 luminal A-like breast cancer is also under investigation, with the EUROPA trial demonstrating equivalent short-term oncologic outcomes but less reduction in health-related quality of life and fewer treatment-related adverse effects with radiotherapy alone compared with sole endocrine therapy.^30^ The increasingly widespread adoption of de-escalation protocols has the potential to lead to a further reduction in treatment-related morbidity, particularly for women with a diagnosis of early-stage disease due to screening participation.

For the purpose of our analysis, we conservatively assumed screen-detected DCIS to be a potential overdiagnosis, but this is often not the case. For this group of middle-aged Australian women with otherwise excellent life expectancy, there is ample time for cases of DCIS or indolent invasive tumors to progress to the point of becoming symptomatic if left untreated. In our cohort, five breast cancer-related deaths occurred among patients defined as potentially overdiagnosed, with no statistically significant difference in breast cancer survival compared with the patients who had screen-detected tumors not considered to be overdiagnosed.

In an analysis of two screening trials, most of the excess DCIS cases detected among women invited to screening were balanced by a later deficit in cases of invasive disease, suggesting a high proportion of progression.^31^ In another study from the UK National Health Service Breast Screening Programme, a significant negative association was found between screen-detection of DCIS and incidence of subsequent interval invasive disease within 3 years.^32^ Although active surveillance of low-risk DCIS is currently under investigation in trials such as COMET^33^ and LORIS,^34^ patients with high-grade disease have been excluded and are likely to remain best served receiving standard therapies.

Screening also detects tumors of limited extent, but nonetheless with aggressive biologic potential. In our cohort, just more than 10% of the screen-detected tumors were HER2-positive, whereas 6% were triple-negative. In an era of precision oncology, identification of such phenotypes at an early stage provides an opportunity to de-escalate systemic therapy. Where neoadjuvant systemic therapy is required, earlier-stage tumors are more likely to achieve a complete pathologic response,^35,36^ translating to a superior prognosis^37^ and less systemic therapy in the adjuvant setting. Systemic therapy also is generally more effective in earlier disease stages due to the smaller tumor burden, perhaps because of less heterogeneity within tumor cells and reduced opportunity to develop mechanisms of resistance to treatment.^38^

## Generalizability

Although we report outcomes from a single breast service, our findings are comparable with national data from a similar period. In 2018, the Australian Institute of Health and Welfare reported that women ages 50–69 years with a diagnosis through BreastScreen Australia between the years 2000 and 2012 had a 77% lower risk of death from breast cancer than those in the same age range with breast cancer who had never been screened.^39^ After model adjustment for potential lead time bias, screen-detected women maintained a 57% lower risk of breast cancer death. National treatment patterns are likely to be similar to those of our study cohort, with Australian national clinical practice guidelines for the management of early-stage breast cancer in existence since 1995.^40^

## Strengths and Limitations

This report describes long-term mortality outcomes for a patient cohort whose detailed treatment has previously been reported and analyzed through linkage with high-quality population cancer, screening, and mortality registry data. Our linkage rate was high (97.3%).

Our analysis included correction for potential bias due to more favorable outcomes among potentially overdiagnosed cancers in AS patients. Although it is not possible to recognize overdiagnosis in an individual, it may be estimated at a population-level based on follow-up data collected over years of screening, or by statistical modeling of disease transition.^41^ Published estimates of overdiagnosis with mammographic screening vary widely, from negligible to just more than 50%.^42^

The Independent UK Panel of Breast Cancer Screening in 2013 sought a best estimate on the basis of follow-up evaluation in three seminal trial populations, finding a 19% overdiagnosis rate during the period of targeted invitation to screening.^3^ Methods that fully account for trends in breast cancer incidence and the effects of lead time lower these estimates to 10% or less.^43,44^

To model the outcomes for the women participating in screening in our cohort, we used a pragmatic approach to calculate overdiagnosis at an individual level, using an a priori definition of disease phenotypes that could be reasonably considered a potential overdiagnosis. We acknowledge that phenotypes outside these criteria also may be potentially overdiagnosed and that some cases considered potential overdiagnoses may not be, depending on individual patient factors such as frailty and comorbidity. Using this somewhat conservative pathologic definition, our rate of overdiagnosis was 32.1% (24.5% in situ, 7.7% invasive). Removing these favorable diagnoses made little difference to the observed survival differences between AS and NRS, confirming that the observed mortality benefits of active screening were not due to overdiagnosis.

We also adjusted our analysis for potential lead time bias, applying a mean lead-time estimate of 40 months to screen-detected cancers, which, although somewhat conservative, has achieved a level of consensus^45^ and also has enabled comparison with national data.^39^

A limitation of this study was that we did not attempt to account for any screening self-selection bias (healthy screener effect) as was observed in some of the seminal screening trials, whereby women who choose to participate in breast cancer screening are at inherently lower risk of death than those who do not participate, even in the absence of a cancer diagnosis. This is difficult to quantify, varying by geographic location, and indeed may be less relevant in an Australian context (which uses universal invitation to screening, at no cost to the individual).^46,47^ In the Australian Institute of Health and Welfare analysis of national data, a realistic correction factor was applied, with minimal change in the estimated hazard of breast cancer death for women with disease diagnosed through BreastScreen Australia.^39^

This study aimed to assess the pragmatic benefit of participating in population-based screening through the BreastScreen Australia program, which historically offered no-cost mammographic screening every 2 years for women ages 50–69 years. The target range was expanded to 74 years from 2013 to 2014, with optional access for women ages 40–49 years and those older than 74 years.^39^

In contrast, the current U.S. guidelines recommend initiating routine screening at the age of 40 years,^48–50^ highlighting variation in international screening practices that may result in different patterns of cancer detection and treatment intensity. In this study, some individuals may have presented to BreastScreen while symptomatic, whereas some defined as NRS may have been engaging in private screening (or represent asymptomatic cancers diagnosed incidentally). Taken together, these effects would dilute our findings, leading to conservative estimates of the benefits from BreastScreen participation. Screening conducted through private providers outside the program may use alternate imaging methods or follow different screening intervals based on individual or clinician preference, introducing additional variability, which could potentially influence our findings.

## Future Work

We included interval cancers within our definition of AS to capture the overall effect of population-based screening for women who chose to participate. In line with previous reports,^51,52^ interval cancers had less favorable pathologic features than screen-detected cancers, with survival outcomes similar to those observed for NRS patients. Future work should therefore focus on the identification of active screeners at higher risk of interval breast cancer who may benefit from alternative screening technologies, screening intervals, or both,^53–55^ aiming to improve the balance of benefits and harms of screening for this population group.

Replicating our analysis in a national cohort with a larger and more diverse population would strengthen the ability to draw more definitive conclusions about any potential associations between screening status and subsequent cancer event outcomes. A broader dataset also would support more nuanced adjustment for the “healthy screener effect” by allowing for incorporation of potential confounding factors such as comorbidity and socioeconomic status.

Larger studies also are needed to examine potential differences in disease subtype-specific survival between AS and NRS. Breast cancer is a heterogeneous spectrum of histogenetic pathways associated with measurable biomarkers that have direct relevance to patient management and disease prognosis. Findings have shown short-term survival outcomes to be considerably lower for patients with triple-negative breast cancer than for patients eligible to receive HER2 targeted or endocrine therapies.^56^ Differential survival by screening status in relation to disease subtype is yet to be evaluated in real-world populations.

## Conclusions

The benefits of population screening include both reduced breast cancer mortality and reduced treatment intensity. Patients whose ESBC is diagnosed while participating in population screening receive less intense treatment and have substantially better BCSS and OS than patients NRS. These findings persist after adjustment for potential overdiagnosis and lead time bias. As treatment for ESBC becomes more personalized, with increasing options for de-escalation of therapy, the benefits of screening are likely to improve further.

## Supplementary Information

Below is the link to the electronic supplementary material.

Supplementary file1 (DOCX 21 KB)

## References

[1] Berry DA, Cronin KA, Plevritis SK, et al. Effect of screening and adjuvant therapy on mortality from breast cancer. *N Engl J Med*. 2005;353:1784–92. 16251534 10.1056/NEJMoa050518

[2] Olsen O, Gøtzsche PC. Screening for breast cancer with mammography. *Cochrane Database Syst Rev.* 2001:CD001877. 10.1002/14651858.CD001877 11687128

[3] Marmot MG, Altman DG, Cameron DA, Dewar JA, Thompson SG, Wilcox M. The benefits and harms of breast cancer screening: an independent review. *Br J Cancer*. 2013;108:2205–40. 23744281 10.1038/bjc.2013.177 PMC3693450

[4] Duffy SW, Yen AM-F, Chen TH-H, et al. Long-term benefits of breast screening. *Breast Cancer Manag*. 2012;1:31–8.

[5] Nelson HD, Fu R, Cantor A, Pappas M, Daeges M, Humphrey L. Effectiveness of breast cancer screening: systematic review and meta-analysis to update the 2009 US Preventive services task force recommendation. *Ann Intern Med*. 2016;164:244–55. 26756588 10.7326/M15-0969

[6] Nyström L, Andersson I, Bjurstam N, Frisell J, Nordenskjöld B, Rutqvist LE. Long-term effects of mammography screening: updated overview of the Swedish randomised trials. *Lancet*. 2002;359:909–19. 11918907 10.1016/S0140-6736(02)08020-0

[7] Lauby-Secretan B, Scoccianti C, Loomis D, et al. Breast-cancer screening: viewpoint of the IARC Working Group. *N Engl J Med*. 2015;372:2353–8. 26039523 10.1056/NEJMsr1504363

[8] Gøtzsche PC, Jørgensen KJ. Screening for breast cancer with mammography. *Cochrane Database Syst Rev.* 2013:CD001877. 10.1002/14651858.CD001877.pub5 PMC6464778 23737396

[9] Bennett A, Shaver N, Vyas N, et al. Screening for breast cancer: a systematic review update to inform the Canadian Task Force on Preventive Health Care guideline. *Syst Rev*. 2024;13:304. 39702409 10.1186/s13643-024-02700-3 PMC11656969

[10] Barratt A. Overdiagnosis in mammography screening: a 45-year journey from shadowy idea to acknowledged reality. *Br Med J*. 2015;350:h867. 25736426 10.1136/bmj.h867

[11] Baum M. Harms from breast cancer screening outweigh benefits if death caused by treatment is included. *Br Med J*. 2013;346:f385. 23344314 10.1136/bmj.f385

[12] Biller-Andorno N, Jüni P. Abolishing mammography screening programs? A view from the Swiss Medical Board. *Obstet Gynecol Surv*. 2014;69:474–5. 10.1056/NEJMp1401875 24738641

[13] Elder K, Nickson C, Pattanasri M, et al. Treatment intensity differences after early-stage breast cancer (ESBC) diagnosis depending on participation in a screening program. *Ann Surg Oncol*. 2018;25:2563–72. 29717421 10.1245/s10434-018-6469-7

[14] Rubin D. Multiple imputation for nonresponse in surveys. Hoboken: Wiley; 1987.

[15] Duffy SW, Nagtegaal ID, Wallis M, et al. Correcting for lead time and length bias in estimating the effect of screen detection on cancer survival. *Am J Epidemiol*. 2008;168:98–104. 18504245 10.1093/aje/kwn120

[16] Welch HG, Frankel BA. Likelihood that a woman with screen-detected breast cancer has had her “life saved” by that screening. *Arch Intern Med*. 2011;171:2043–6. 22025097 10.1001/archinternmed.2011.476

[17] Wilkinson AN, Mainprize JG, Yaffe MJ, et al. Cost-Effectiveness of breast cancer screening using digital mammography in Canada. *JAMA Netw Open*. 2025;8:e2452821. 39745700 10.1001/jamanetworkopen.2024.52821 PMC11696453

[18] Dempsey K, Costa DS, Brennan ME, Mann GB, Snook KL, Spillane AJ. Benefits and harms of breast cancer screening revisited: a large, retrospective cross-sectional study quantifying treatment intensity in women with screen-detected versus non-screen-detected cancer in Australia and New Zealand. *BMJ Oncol*. 2023;2:e000100. 39886492 10.1136/bmjonc-2023-000100 PMC11203081

[19] Spillane AJ, Kennedy CW, Gillett DJ, et al. Screen-detected breast cancer compared to symptomatic presentation: an analysis of surgical treatment and end-points of effective mammographic screening. *ANZ J Surg*. 2001;71:398–402. 11450913 10.1046/j.1440-1622.2001.02144.x

[20] Gentilini OD, Botteri E, Sangalli C, et al. Sentinel lymph node biopsy vs no axillary surgery in patients with small breast cancer and negative results on ultrasonography of axillary lymph nodes: the SOUND randomized clinical trial. *JAMA Oncol*. 2023;9:1557–64. 37733364 10.1001/jamaoncol.2023.3759 PMC10514873

[21] Reimer T, Stachs A, Veselinovic K, et al. Axillary surgery in breast cancer: primary results of the INSEMA trial. *N Engl J Med*. 2024;392:1051–64. 39665649 10.1056/NEJMoa2412063

[22] Hughes KS, Schnaper LA, Bellon JR, et al. Lumpectomy plus tamoxifen with or without irradiation in women age 70 years or older with early breast cancer: long-term follow-up of CALGB 9343. *J Clin Oncol*. 2013;31:2382–7. 23690420 10.1200/JCO.2012.45.2615 PMC3691356

[23] Kunkler IH, Williams LJ, Jack WJ, Cameron DA, Dixon JM. Breast-conserving surgery with or without irradiation in early breast cancer. *N Engl J Med*. 2023;388:585–94. 36791159 10.1056/NEJMoa2207586

[24] Whelan TJ, Smith S, Parpia S, et al. Omitting radiotherapy after breast-conserving surgery in luminal A breast cancer. *N Engl J Med*. 2023;389:612–9. 37585627 10.1056/NEJMoa2302344

[25] Chua B, Gray K, Krishnasamy M, et al. Abstract OT2-04-03: examining personalized radiation therapy (EXPERT): a randomised phase III trial of adjuvant radiotherapy vs observation in patients with molecularly characterized luminal A breast cancer. *Cancer Res*. 2019;79(4 Suppl):0T2-04–03.

[26] White JR, Anderson SJ, Harris EE, et al. NRG-BR007: a phase III trial evaluating de-escalation of breast radiation (DEBRA) following breast-conserving surgery (BCS) of stage 1, hormone receptor+, HER2–, RS≤ 18 breast cancer. *J Clin Oncol*. 2022;40(16 Suppl):TPS613.

[27] Braunstein LZ, Wong J, Dillon DA, et al. Abstract OT1-12-02: preliminary report of the PRECISION trial (profiling early breast cancer for radiotherapy omission): a phase II study of breast-conserving surgery without adjuvant radiotherapy for favorable-risk breast cancer. *Cancer Res*. 2023;83:OT1-12–02.

[28] Jagsi R, Griffith KA, Harris EE, et al. Omission of radiotherapy after breast-conserving surgery for women with breast cancer with low clinical and genomic risk: 5-year outcomes of IDEA. *J Clin Oncol*. 2024;42:390–8. 38060195 10.1200/JCO.23.02270 PMC11846025

[29] Mann GB, Skandarajah AR, Zdenkowski N, et al. Postoperative radiotherapy omission in selected patients with early breast cancer following preoperative breast MRI (PROSPECT): primary results of a prospective two-arm study. *Lancet*. 2024;403:261–70. 38065194 10.1016/S0140-6736(23)02476-5

[30] Meattini I, De Santis MC, Visani L, et al. Single-modality endocrine therapy versus radiotherapy after breast-conserving surgery in women aged 70 years and older with luminal A-like early breast cancer (EUROPA): a preplanned interim analysis of a phase 3, non-inferiority, randomised trial. *Lancet Oncol*. 2025;26:37–50. 39675376 10.1016/S1470-2045(24)00661-2

[31] Duffy SW, Agbaje O, Tabar L, et al. Overdiagnosis and overtreatment of breast cancer: estimates of overdiagnosis from two trials of mammographic screening for breast cancer. *Breast Cancer Res*. 2005;7:258–65. 16457701 10.1186/bcr1354 PMC1410738

[32] Duffy SW, Dibden A, Michalopoulos D, et al. Screen detection of ductal carcinoma *in situ* and subsequent incidence of invasive interval breast cancers: a retrospective population-based study. *Lancet Oncol*. 2016;17:109–14. 26655422 10.1016/S1470-2045(15)00446-5 PMC4691349

[33] Hwang ES, Hyslop T, Lynch T, et al. Active monitoring with or without endocrine therapy for low-risk ductal carcinoma *in situ:* the COMET randomized clinical trial. *JAMA*. 2025;333:972–80. 39665585 10.1001/jama.2024.26698 PMC11920841

[34] Francis A, Thomas J, Fallowfield L, et al. Addressing overtreatment of screen detected DCIS; the LORIS trial. *Eur J Cancer*. 2015;51:2296–303. 26296293 10.1016/j.ejca.2015.07.017

[35] Goorts B, van Nijnatten TJ, de Munck L, et al. Clinical tumor stage is the most important predictor of pathological complete response rate after neoadjuvant chemotherapy in breast cancer patients. *Breast Cancer Res Treat*. 2017;163:83–91. 28205044 10.1007/s10549-017-4155-2 PMC5387027

[36] Livingston-Rosanoff D, Schumacher J, Walle KV, et al. Does tumor size predict response to neoadjuvant chemotherapy in the modern era of biologically driven treatment? A nationwide study of US breast cancer patients. *Clin Breast Cancer*. 2019;19:e741–7. 31300338 10.1016/j.clbc.2019.05.014 PMC6888946

[37] Asselain B, Barlow W, Bartlett J, et al. Long-term outcomes for neoadjuvant versus adjuvant chemotherapy in early breast cancer: meta-analysis of individual patient data from ten randomised trials. *Lancet Oncol*. 2018;19:27–39. 29242041 10.1016/S1470-2045(17)30777-5 PMC5757427

[38] Ellsworth RE, Blackburn HL, Shriver CD, Soon-Shiong P, Ellsworth DL. Molecular heterogeneity in breast cancer: state of the science and implications for patient care. *Semin Cell Dev Biol*. 2017;64:65–72. 27569190 10.1016/j.semcdb.2016.08.025

[39] Australian Institute of Health and Welfare. Analysis of cancer outcomes and screening behaviour for national cancer screening programs in Australia. Cancer series no. 111. Cat. no. CAN 115. Canberra: AIHW; 2018.

[40] National Health and Medical Research Council. Clinical practice guidelines for the management of early breast cancer. Canberra: Australian Government Publishing Service; 1995 [rescinded in March 2003 after publication of the 2nd edition in 2001].

[41] Biesheuvel C, Barratt A, Howard K, Houssami N, Irwig L. Effects of study methods and biases on estimates of invasive breast cancer overdetection with mammography screening: a systematic review. *Lancet Oncol*. 2007;8:1129–38. 18054882 10.1016/S1470-2045(07)70380-7

[42] Chaltiel D, Hill C. Estimations of overdiagnosis in breast cancer screening vary between 0% and over 50%: why? *BMJ Open*. 2021;11:e046353. 34158298 10.1136/bmjopen-2020-046353 PMC8220464

[43] Puliti D, Duffy S, Miccinesi G, et al. Overdiagnosis in mammographic screening for breast cancer in Europe: a literature review. *J Med Screen*. 2012;19:42–56. 22972810 10.1258/jms.2012.012082

[44] Duffy SW, Tabar L, Olsen AH, et al. Absolute numbers of lives saved and overdiagnosis in breast cancer screening, from a randomized trial and from the breast screening programme in England. *J Med Screen*. 2010;17:25–30. 20356942 10.1258/jms.2009.009094 PMC3104821

[45] Duffy SW, Parmar D. Overdiagnosis in breast cancer screening: the importance of length of observation period and lead time. *Breast Cancer Res*. 2013;15:R41. 23680223 10.1186/bcr3427 PMC3706885

[46] Roder D, Houssami N, Farshid G, et al. Population screening and intensity of screening are associated with reduced breast cancer mortality: evidence of efficacy of mammography screening in Australia. *Breast Cancer Res Treat*. 2008;108:409–16. 18351455 10.1007/s10549-007-9609-5

[47] Nickson C, Mason KE, English DR, Kavanagh AM. Mammographic screening and breast cancer mortality: a case–control study and meta-analysis. *Cancer Epidemiol Biomarkers Prev*. 2012;21:1479–88. 22956730 10.1158/1055-9965.EPI-12-0468

[48] Nicholson WK, Silverstein M, Wong JB, et al. Screening for breast cancer: US preventive services task force recommendation statement. *JAMA*. 2024;331:1918–30. 38687503 10.1001/jama.2024.5534

[49] Monticciolo DL, Newell MS, Hendrick RE, et al. Breast cancer screening for average-risk women: recommendations from the ACR commission on breast imaging. *J Am Coll Radiol*. 2017;14:1137–43. 28648873 10.1016/j.jacr.2017.06.001

[50] Chen M, Hill CC, Zaritsky E. Age to initiate routine breast cancer screening: ACOG clinical practice update. *Obstet Gynecol*. 2025;145:e40–4. 39388713 10.1097/AOG.0000000000005757

[51] Niraula S, Biswanger N, Hu P, Lambert P, Decker K. Incidence, characteristics, and outcomes of interval breast cancers compared with screening-detected breast cancers. *JAMA Netw Open*. 2020;3:e2018179. 32975573 10.1001/jamanetworkopen.2020.18179 PMC7519419

[52] McCarthy AM, Barlow WE, Conant EF, et al. Breast cancer with a poor prognosis diagnosed after screening mammography with negative results. *JAMA Oncol*. 2018;4:998–1001. 29801067 10.1001/jamaoncol.2018.0352 PMC6145719

[53] Bakker MF, de Lange SV, Pijnappel RM, et al. Supplemental MRI screening for women with extremely dense breast tissue. *N Engl J Med*. 2019;381:2091–102. 31774954 10.1056/NEJMoa1903986

[54] Salim M, Liu Y, Sorkhei M, et al. AI-based selection of individuals for supplemental MRI in population-based breast cancer screening: the randomized ScreenTrustMRI trial. *Nat Med*. 2024;30:2623–30. 38977914 10.1038/s41591-024-03093-5 PMC11405258

[55] The Daffodil Centre. The ROSA Project–summary: summary of “Roadmap for Optimising Screening in Australia–Breast,” investigating risk-based breast cancer screening. Produced by the Daffodil Centre on behalf of Cancer Council Australia. 2023.

[56] Howlader N, Cronin KA, Kurian AW, Andridge R. Differences in breast cancer survival by molecular subtypes in the United States. *Cancer Epidemiol Biomarkers Prev*. 2018;27:619–26. 29593010 10.1158/1055-9965.EPI-17-0627

