# Supplementary material for: Survival Benefits and Less Intensive Treatment for Women with Early-Stage Breast Cancer Diagnosed While Participating in Population-Based Screening
Source: Ann Surg Oncol. 2025 Jul 25;32(11):8100–10. doi: 10.1245/s10434-025-17845-1 (PMC12494613; doi:10.1245/s10434-025-17845-1)
Supplement: Supplementary file 1 — Supplementary file1 (DOCX 21 KB) [file 10434_2025_17845_MOESM1_ESM.docx]

**Supplementary Table 1. Characteristics of potentially overdiagnosed versus not overdiagnosed screen-detected cases**

|  | **Overdiagnosed** | | **Not overdiagnosed screen-detected** | | **P value** |
| --- | --- | --- | --- | --- | --- |
|  | ***N*** | **%** | ***N*** | **%** |  |
| ***N*** | 180 | 32.1 | 380 | 67.9 |  |
| **Age, years** |  |  |  |  |  |
| 50-59 | 77 | 42.8 | 189 | 49.7 | 0.12 |
| 60-69 | 103 | 57.2 | 191 | 50.3 |  |
| **Menopausal status** |  |  |  |  |  |
| Pre/peri-menopausal | 24 | 13.3 | 44 | 11.6 | 0.02 |
| Post-menopausal | 140 | 77.8 | 267 | 70.3 |  |
| Unknown | 16 | 8.89 | 69 | 18.2 |  |
| **Invasive/*in situ*** |  |  |  |  |  |
| Invasive | 43 | 23.9 | 380 | 100 | <0.001 |
| *In situ* | 137 | 76.1 | 0 | 0 |  |
| **Size (invasive)**  Median (IQR) | 6 (5-8) | | 14 (10-20) | | <0.001 |
| <10mm | 43 | 100 | 87 | 22.9 |  |
| 10-19mm | 0 | 0 | 188 | 49.5 |  |
| 20-29mm | 0 | 0 | 65 | 17.1 |  |
| ≥30mm | 0 | 0 | 40 | 10.5 |  |
| **Grade (invasive)** |  |  |  |  |  |
| 1 | 43 | 100 | 65 | 17.1 | <0.001 |
| 2 | 0 | 0 | 168 | 44.2 |  |
| 3 | 0 | 0 | 112 | 29.5 |  |
| Unknown | 0 | 0 | 35 | 9.21 |  |
| **ER status** |  |  |  |  |  |
| Positive | 152 | 84.4 | 340 | 89.5 | <0.001 |
| Negative | 18 | 10.0 | 40 | 10.5 |  |
| Unknown | 10 | 5.56 | 0 | 0 |  |
| **PR status** |  |  |  |  |  |
| Positive | 130 | 72.2 | 301 | 79.2 | <0.001 |
| Negative | 40 | 22.2 | 78 | 20.5 |  |
| Unknown | 10 | 5.56 | 1 | 0.26 |  |
| **Breast surgery** |  |  |  |  |  |
| BCS | 148 | 82.2 | 329 | 86.6 | 0.55 |
| Mastectomy | 32 | 17.8 | 60 | 15.8 |  |
| **Axillary surgery** |  |  |  |  |  |
| SLNB | 95 | 52.8 | 303 | 79.7 | <0.001 |
| ALND (+/- SLNB) | 3 | 1.67 | 71 | 18.7 |  |
| None | 82 | 45.6 | 6 | 1.58 |  |
| **Radiation therapy**^a^ |  |  |  |  |  |
| No | 86 | 47.8 | 73 | 19.2 | <0.001 |
| Yes | 94 | 52.2 | 307 | 80.8 |  |
| **Chemotherapy**^a^ |  |  |  |  |  |
| No | 179 | 99.4 | 241 | 63.4 | <0.001 |
| Yes | 1 | 0.56 | 139 | 36.6 |  |
| **Endocrine therapy**^a^ |  |  |  |  |  |
| No | 57 | 31.7 | 52 | 13.7 | <0.001 |
| Yes | 123 | 68.3 | 328 | 86.3 |  |
| **Subsequent cancer events** |  |  |  |  |  |
| Ipsilateral breast events | 15 | 8.33 | 13 | 3.42 | 0.013 |
| Contralateral breast events | 10 | 5.56 | 19 | 5.00 | 0.78 |
| **Deaths** |  |  |  |  |  |
| Breast cancer | 5 | 2.78 | 20 | 5.26 | 0.85 |
| Other cancer | 3 | 1.67 | 14 | 3.68 |  |
| Non-cancer | 8 | 4.44 | 23 | 6.05 |  |
| Unknown | 0 | 0 | 1 | 0.26 |  |

^a^ Treatment recommendation from multidisciplinary meeting.

Overdiagnosis defined as screen-detected DCIS or screen-detected grade 1, <10mm, ER and/or PR positive, Her2 negative invasive cancers.

Abbreviations: ALND=axillary lymph node dissection, BCS=breast conserving surgery, ER=estrogen receptor, IQR=interquartile range, PR=progesterone receptor, SLNB=sentinel lymph node biopsy.
